# Association of Real-Time Glucose Levels and Cognitive Performance in Children with Type 1 Diabetes: A Cross-Sectional Study

**DOI:** 10.3390/jcm15187080

**Published:** 2026-09-12

**Authors:** Paulina Wais, Maia Stanisławska-Kubiak, Elżbieta Niechciał, Piotr Fichna, Andrzej Kędzia, Ewa Mojs, Katarzyna Anna Majewska

**Affiliations:** 1 Department of Pediatric Diabetes, Auxology and Obesity, Poznan University of Medical Sciences, 60-572 Poznan, Poland; eniechcial@ump.edu.pl (E.N.); pfichna@ump.edu.pl (P.F.); akedzia@ump.edu.pl (A.K.); 2 Department of Clinical Psychology, Poznan University of Medical Sciences, 60-812 Poznan, Poland; maiakubiak@ump.edu.pl (M.S.-K.); ewamojs@ump.edu.pl (E.M.)

**Keywords:** type 1 diabetes, attention, cognitive functions, glycemia, children

## Abstract

**Background/Objectives**: Subtle cognitive alterations have been described in children with type 1 diabetes (T1D), but the association between cognition and real-time glycemia during cognitive testing remains unclear. This study aimed to investigate whether blood glucose level (BGL) measured immediately before the MOXO Continuous Performance Test (MOXO-CPT) is associated with cognitive performance and modified by clinical factors. **Methods**: A total of 117 children with T1D (aged 6–18 years) completed the MOXO-CPT assessing sustained attention, timeliness, impulsivity, and hyperactivity. Participants were stratified by BGL measured immediately before testing (<200 mg/dL [<11.1 mmol/L] vs. ≥200 mg/dL [≥11 mmol/L]). Group comparisons were performed using the Mann–Whitney U test. Multivariable linear regression models examined associations between BGL and cognitive outcomes, adjusting for HbA1c, age, sex, treatment modality and diabetes duration. Interaction analyses were also conducted. **Results:** In unadjusted group comparisons, participants with BGL ≥ 200 mg/dL demonstrated nominally higher sustained attention (*p* = 0.036) and timeliness scores (*p* = 0.040) than those with BGL < 200 mg/dL. However, these differences did not remain statistically significant after Benjamini–Hochberg correction and were not confirmed after adjustment for relevant covariates. No differences were observed in impulsivity or hyperactivity. In multivariable models, higher BGLs were independently associated with higher impulsivity scores (B = 0.013, *p* = 0.007), with higher scores indicating better impulse-control performance. A significant BGL–sex interaction was observed for timeliness. Sex-stratified analyses showed that higher BGLs were associated with better attention and timeliness in girls, whereas the association with impulsivity observed in boys was not confirmed using robust analysis. No significant interactions between BGL and HbA1c, age, treatment modality, or diabetes duration were observed after robust analysis. **Conclusions**: Real-time glycemia during cognitive testing was associated with subtle, domain-specific differences in cognitive performance in children with T1D. These findings do not establish causality or indicate that higher glucose levels improve cognitive performance. Concurrent glucose levels may be worth considering when interpreting cognitive assessments, although this should be confirmed in prospective studies.

## 1. Introduction

Type 1 diabetes (T1D) is one of the most common chronic diseases of childhood and has been associated with subtle but measurable alterations in neurocognitive functioning. Children with T1D may exhibit mild deficits in attention, processing speed, and executive functioning compared with healthy peers [1,2,3]. A meta-analysis of 19 studies including 1355 children and adolescents with T1D and 696 healthy controls found significantly poorer cognitive performance in youth with T1D, with particularly pronounced differences in attention (Hedges’ g = −0.60) and psychomotor speed (Hedges’ g = −0.46) [4]. These alterations are often subtle and may not be evident in routine clinical assessments; however, they may have important implications for academic performance and daily functioning. Most previous studies have focused on chronic metabolic indices, such as HbA1c or cumulative glycemic exposure, which have been linked to altered cognitive trajectories, brain development, and neurodevelopmental processes during critical periods of brain maturation [5,6]. In addition, previous research has investigated the impact of acute metabolic complications, such as hypoglycemia and diabetic ketoacidosis (DKA), on cognitive functioning in children with T1D [7,8]. Hypoglycemia has been associated with transient impairments in attention and executive functioning [7], whereas DKA, particularly when occurring early during T1D, has been associated with alterations in brain development and neurocognitive outcomes [8]. However, the short-term effects of real-time glycemia during cognitive task performance remain less well understood. Previous experimental and clinical studies suggest that cognitive performance may be influenced by acute glucose fluctuations [7,9,10]. Specifically, hypoglycemia may impair executive functioning [7,11], while hyperglycemia may affect attentional processes [9,12], although these effects are not necessarily linear and may depend on individual clinical characteristics [7,13].

Continuous Performance Tests (CPTs), such as the MOXO-CPT, provide objective measures of sustained attention, timing, and response control [14]. In the present study, the four MOXO-CPT parameters were sustained attention, timeliness, hyperactivity, and impulsivity. These tools allow for the assessment of domain-specific cognitive processes under standardized conditions and may be particularly sensitive to subtle fluctuations in cognitive efficiency. Despite their widespread use, the potential association between concurrent glycemia during test administration and these cognitive domains has not been sufficiently investigated in pediatric T1D populations. Therefore, the aim of this study was to assess whether blood glucose levels (BGLs) measured during MOXO-CPT performance are associated with cognitive outcomes in children and adolescents with T1D.

## 2. Materials and Methods

### 2.1. Study Design and Participants

This study was conducted as part of a larger research project involving children and adolescents with T1D. A total of 117 patients aged 6–18 years were included. Participants were recruited consecutively between December 2019 and December 2021, during routine clinical visits at the Department of Pediatric Diabetes, Auxology and Obesity, Poznan University of Medical Sciences. Inclusion criteria comprised a confirmed diagnosis of T1D and an age between 6 and 18 years. Exclusion criteria comprised hypoglycemia during testing (BGL < 70 mg/dL [<3.9 mmol/L]), or concerning symptoms of poor well-being, including symptoms suggestive of hypoglycemia, as well as neurological disorders, psychiatric conditions, severe comorbidities, and lack of informed consent. All participants were assessed under stable clinical conditions. Written informed consent was obtained from parents or legal guardians of all participants, as well as from participants aged ≥16 years. The study was conducted in accordance with the Declaration of Helsinki and approved by the Bioethics Committee at Poznan University of Medical Sciences.

### 2.2. Clinical and Metabolic Data

Clinical data were obtained from medical records and included: age, sex, duration of diabetes, glycated hemoglobin (HbA1c), treatment modality (multiple daily injections [MDI] or continuous subcutaneous insulin infusion [CSII]). BGLs were measured immediately before the cognitive assessment using a capillary blood sample with the MultiSure GK blood glucose meter (Apex Biotechnology Corp., Hsinchu, Taiwan). The MOXO-CPT was initiated immediately after the glucose measurement. Time since the last meal and time since the last insulin dose were not recorded. For analysis, participants were stratified into two groups based on glucose level: group I with a BGL < 200 mg/dL [<11.1 mmol/L] and group II with a BGL ≥ 200 mg/dL [≥11.1 mmol/L]. A threshold of 200 mg/dL was used as a clinically pragmatic indicator of marked above-target glycemia during cognitive testing rather than as a validated neurocognitive threshold. The selected cut-off aligns with International Society for Pediatric and Adolescent Diabetes (ISPAD) criteria defining blood glucose levels ≥ 200 mg/dL (11.1 mmol/L) as clinically significant hyperglycemia [15].

### 2.3. Cognitive Assessment

Attentional functioning and related aspects of executive control were assessed using the MOXO Continuous Performance Test (MOXO-CPT), a computerized neuropsychological tool belonging to the class of Continuous Performance Tests (CPTs),developed by Neuro-Technology Solutions, Ltd. (software/report version V6.3.01). The MOXO-CPT is designed to provide an objective and standardized evaluation of attention and behavioral regulation, minimizing subjective bias by measuring real-time responses to stimuli. The test has been validated for use in both clinical and research settings and enables comparison of individual performance with age- and sex-adjusted normative data derived from typically developing individuals. The normative database is international and multicultural. Therefore, the resulting Z-scores were interpreted relative to the MOXO-CPT reference population, while acknowledging that these norms have not been specifically validated in a Polish pediatric cohort. The normative database is international and multicultural. It is available in two versions. A pediatric version for children aged 6–12 years and an adolescent/adult version for individuals aged 13–65 years. The test duration is approximately 15 min for children and 18–18.5 min for older participants [13,15]. In the present study, the pediatric version was used for participants aged 6–12 years and the adolescent/adult version for participants aged 13–18 years, with Z-scores derived using the age- and sex-adjusted normative data corresponding to the respective version.

The MOXO-CPT was administered individually using a computer interface under standardized conditions. Prior to the test, participants received instructions and completed a short practice session to ensure understanding of the task requirements. During the task, participants were instructed to respond as quickly and accurately as possible to a predefined target stimulus by pressing a designated key, while refraining from responding to non-target stimuli. The task requires continuous monitoring of stimuli and inhibition of inappropriate responses, thereby engaging sustained attention and executive control processes. The MOXO-CPT consists of eight sequential stages, each differing in the type, modality, and intensity of three types of distractors: visual, auditory, and combined audiovisual. These distractors are introduced to simulate real-life environmental conditions and increase the ecological validity of the assessment [14,16,17].

The MOXO-CPT provides four primary indices reflecting different components of attentional functioning: sustained attention (A), the ability to correctly identify and respond to target stimuli; timeliness (T), the speed and accuracy of responses within the required time frame; impulsivity (I), responses made prematurely or inappropriately to non-target stimuli; and hyperactivity (H), excessive or irrelevant motor responses during task performance. Each index represents a distinct cognitive domain, allowing for a multidimensional assessment of attentional processes [14,17,18]. For all four indices, higher standardized scores indicate better performance; thus, higher impulsivity and hyper-reactivity scores reflect fewer impulsive or excessive responses.

Responses are classified based on their accuracy and timing. Correct responses to target stimuli within the appropriate time window are recorded as accurate attention and timing performance. Responses to non-target stimuli are classified as impulsivity, while excessive or repeated responses beyond task requirements are categorized as hyperactivity.

The scoring system differentiates between correct responses, omissions, commission errors, and additional responses, forming the basis for calculating the four core indices [18].

Raw scores obtained in each domain were standardized using age- and sex-adjusted normative data and expressed as Z-scores, representing the number of standard deviations from the population mean. Based on these values, performance was classified into four categories: Z ≥ 0 indicating above-average performance, −0.825 ≤ Z < 0 reflecting average performance, −1.65 ≤ Z < −0.825 indicating below-average performance, and Z < −1.65 suggesting clinically significant difficulties. For scores falling below the normative range (Z < −1.65), additional severity levels were applied to further characterize the extent of impairment. Higher scores reflected better cognitive performance [14].

**Figure 1:**
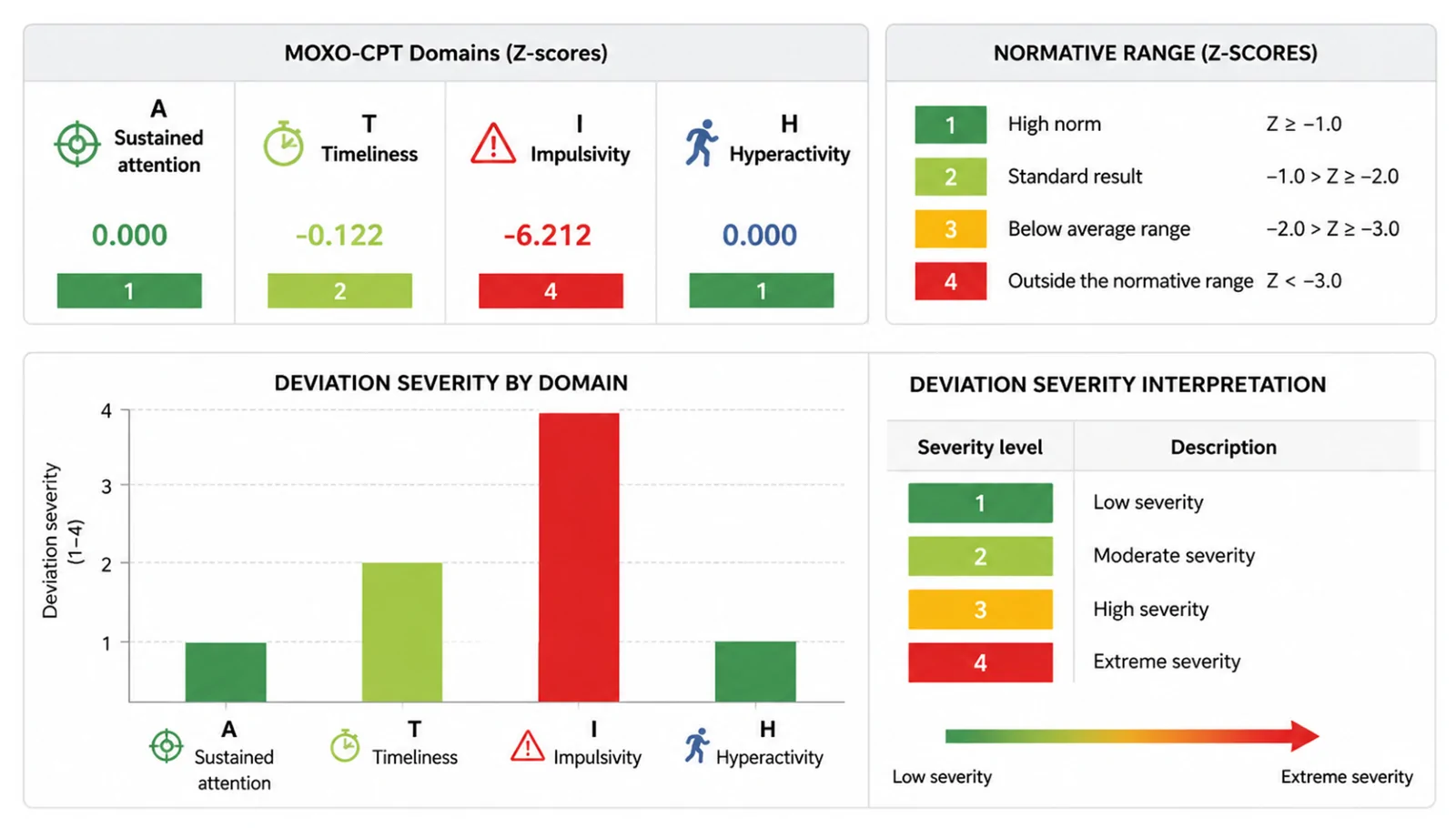
Illustrative MOXO-CPT participant profile and normative comparison (Z-scores). Note: The figure presents an illustrative example of an individual MOXO-CPT participant result and demonstrates the presentation of results across the four MOXO-CPT domains. It is not intended to represent typical or average performance in the study population. Source: Own elaboration.

Furthermore, the MOXO-CPT provides a detailed performance profile across different stages of the test, enabling analysis of variability in attention under varying distractor conditions. This allows for the identification of specific cognitive vulnerabilities, such as increased sensitivity to auditory or visual interference. An illustrative example of a participant’s MOXO-CPT profile is presented in Figure 1.

### 2.4. Statistical Analysis

Statistical analyses were performed using IBM SPSS Statistics (version 29). Continuous variables are presented as mean ± standard deviation (SD), and categorical variables as counts and percentages. Group comparisons were performed using the Mann–Whitney U test for continuous variables (due to non-normal distribution) and the Chi-square test for categorical variables. Effect sizes for nonparametric tests were expressed as r. The prespecified primary analysis compared MOXO-CPT performance between participants with BGLs < 200 mg/dL and those with BGLs ≥ 200 mg/dL. BGL was additionally analyzed as a continuous exposure in multivariable regression models to examine associations across the observed glucose range.

Associations between BGL and cognitive outcomes were further examined using linear regression models, adjusted for HbA1c, age, sex, type of treatment, and duration of diabetes. Diabetes duration was entered into the regression models as a dichotomous variable, coded as 1 for ≤5 years and 2 for >5 years. Statistical significance was set at *p* < 0.05. Sex and diabetes duration were not prespecified as effect modifiers; therefore, interaction and sex-stratified analyses were considered exploratory and hypothesis-generating and were not specifically powered. To account for multiple testing, the Benjamini–Hochberg procedure was applied separately to the four domain-specific comparisons within each analysis family, including the overall glycemic-group comparison, sex-stratified glycemic-group comparisons, sex comparisons within each glycemic group, and the four domain-specific multivariable BGL associations. The potential for type I error was considered when interpreting the findings. Model assumptions were assessed using residual diagnostics, including assessment of residual distribution, homoscedasticity, and influential observations. Because heteroscedasticity was identified in the impulsivity model, HC3 heteroscedasticity-robust standard errors were used as a sensitivity analysis. Sensitivity analyses were also performed after excluding observations with a Cook’s distance > 4/*n*. No formal a priori sample-size calculation was performed; the sample size was determined by the number of eligible participants with complete data available for the present analysis. All 117 participants included in the analysis had complete data for the cognitive outcomes and covariates included in the respective models. No missing-data imputation was performed. The main multivariable models included six predictors and were fitted in 117 participants. Sex-stratified models included five predictors and were fitted in 55 girls and 62 boys, while interaction models included seven predictors and were fitted in the full sample.

## 3. Results

A total of 117 children and adolescents with T1D were included in the analysis. Patients were stratified according to BGL into two groups: group I with glucose < 200 mg/dL (<11.1 mmol/L) (*n* = 93) and group II with glucose ≥ 200 mg/dL (≥11.1 mmol/L) (*n* = 24). There were no significant differences between the groups in terms of age, sex distribution, HbA1c levels, diabetes duration, or treatment modality (all *p* > 0.05). As expected, the groups differed significantly in blood glucose levels measured before testing (*p* < 0.001). In group I, the mean BGL was 127.1 ± 33.7 mg/dL (7.05 ± 1.87 mmol/L), whereas in group II, it was markedly higher, reaching 236.2 ± 34.0 mg/dL (13.11 ± 1.89 mmol/L). Patient clinical and demographic characteristics are presented in Table 1.

### 3.1. Comparison of MOXO-CPT Performance According to Glycemia

**Figure 2:**
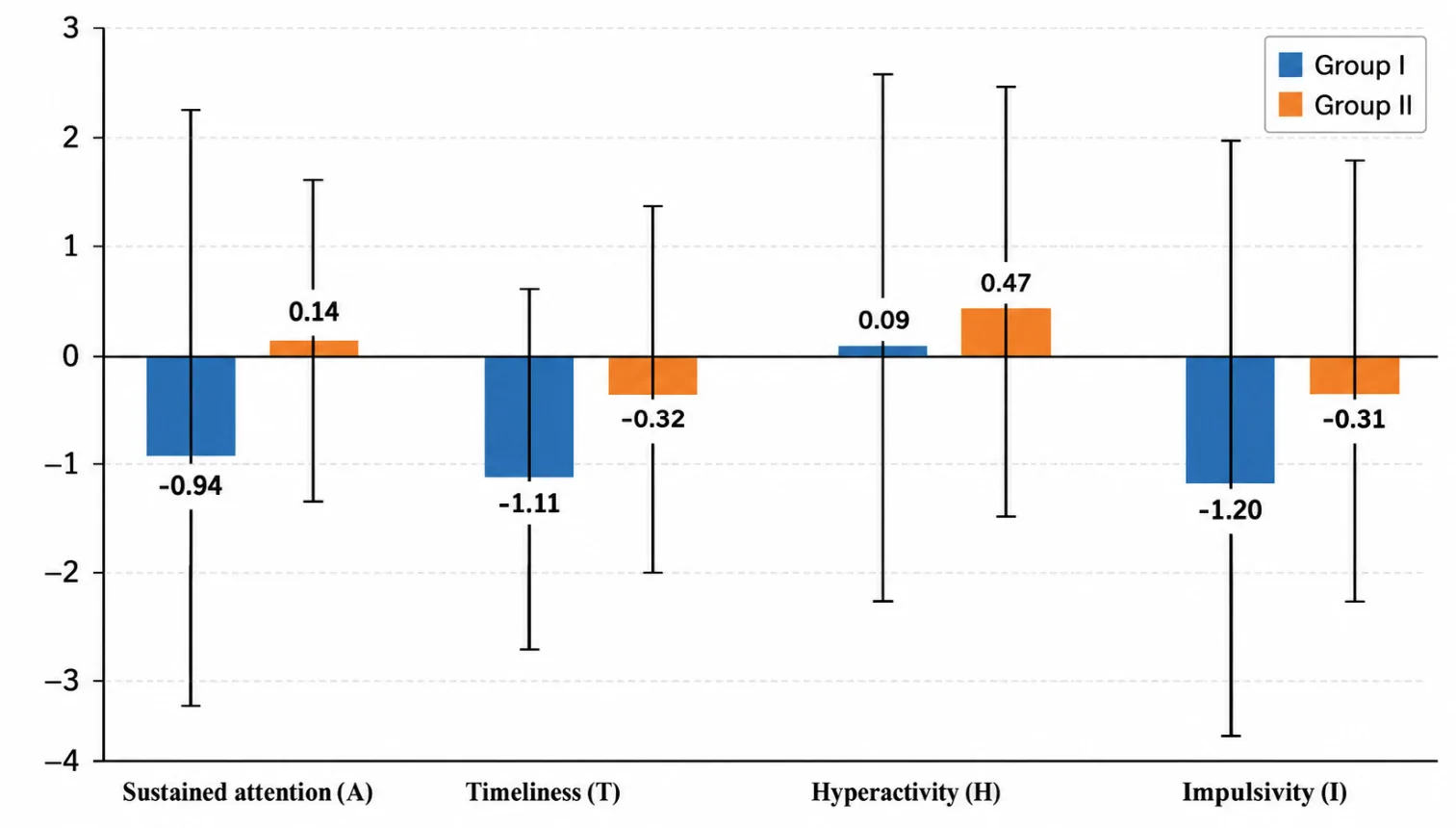
Comparison of MOXO-CPT parameters according to blood glucose level during the test. Note: Group I, BGL < 200 mg/dL (<11.1 mmol/L); group II, BGL ≥ 200 mg/dL (≥11.1 mmol/L). Error bars are 95% confidence intervals of the mean results.

Comparisons of MOXO-CPT outcomes between groups were performed using the Mann–Whitney U test due to the non-normal distribution of variables. In the unadjusted group comparisons, nominally significant differences were observed in sustained attention and timeliness. Patients in group II demonstrated higher scores compared with those in group I in sustained attention (Z = 2.10, *p* = 0.036, r = 0.28) and timeliness (Z = 2.05, *p* = 0.040, r = 0.27), corresponding to small-to-moderate effect sizes. However, after Benjamini–Hochberg correction for multiple comparisons, these differences were no longer statistically significant (adjusted *p* = 0.080 for both). These group-level differences were also not confirmed in the adjusted multivariable regression models. No significant differences were observed for hyperactivity or impulsivity (both *p* > 0.05) (Table 2, Figure 2).

### 3.2. Sex-Stratified Analyses

Sex-stratified analyses were considered exploratory and hypothesis-generating. Among girls, participants with BGLs ≥ 200 mg/dL (≥11.1 mmol/L) had higher scores in sustained attention (*p* = 0.003, BH-adjusted *p* = 0.012, r = 0.58) and timeliness (*p* = 0.009, BH-adjusted *p* = 0.018, r = 0.51) compared with those with BGLs < 200 mg/dL. These associations remained statistically significant after Benjamini–Hochberg correction; however, they should be interpreted cautiously given the small size of the hyperglycemia subgroup. No significant differences were observed for hyperactivity or impulsivity in girls (Table 3). No significant differences between glycemic groups were found in boys across any MOXO-CPT domains (all *p* > 0.05). The results are presented in Table 4.

Additional analyses were performed to compare cognitive performance between girls and boys within each glycemic category across all MOXO-CPT domains. In group I, no significant differences between girls and boys were observed in sustained attention (*p* = 0.565), timeliness (*p* = 0.956), impulsivity (*p* = 0.178), or hyperactivity (*p* = 0.769). In group II, girls had higher scores than boys in sustained attention (*p* = 0.022, BH-adjusted *p* = 0.044, r = 0.45) and timeliness (*p* = 0.008, BH-adjusted *p* = 0.032, r = 0.52). No significant sex differences were observed for impulsivity or hyperactivity. These findings were considered exploratory and hypothesis-generating. The results are presented in Table 5.

Separate multivariable linear regression models were fitted for each MOXO-CPT domain, adjusting for HbA1c, age, sex, treatment method, and diabetes duration. The group-level differences observed in the unadjusted analyses for sustained attention and timeliness were not confirmed in the adjusted regression models. In these models, higher BGLs during testing were significantly associated with higher impulsivity scores (B = 0.013, 95% CI: 0.004–0.022, *p* = 0.007, Benjamini–Hochberg-adjusted *p* = 0.028), where higher scores indicated fewer impulsive responses. Each 1 mg/dL increase in blood glucose level was associated with a 0.013-point increase in impulsivity score. Across the group mean BGLs (127.1 and 236.2 mg/dL), this coefficient corresponded to an estimated difference of approximately 1.4 points in the impulsivity Z-score.

Model diagnostics identified heteroscedasticity in the impulsivity model; therefore, HC3 robust standard errors were used as a sensitivity analysis. The association between BGL and impulsivity remained statistically significant using robust standard errors (B = 0.013, *p* = 0.029). In a further sensitivity analysis excluding observations with Cook’s distance > 4/*n*, the association remained significant (B = 0.008, *p* = 0.036), indicating that the association was not fully explained by influential observations.

Additionally, a higher HbA1c was associated with lower impulsivity scores (B = −0.653, 95% CI: −1.017 to −0.289, *p* < 0.001). Participants with diabetes durations > 5 years had higher impulsivity scores than those with diabetes durations ≤ 5 years (B = 1.553, 95% CI: 0.344 to 2.762, *p* = 0.012), with higher scores indicating fewer impulsive responses. No significant associations between BGL, HbA1c, age, sex, type of treatment, or diabetes duration and performance in sustained attention, timeliness, or hyperactivity were observed in the regression models (all *p* > 0.05). The adjusted R^2^ values for the models predicting sustained attention, timeliness, hyperactivity, and impulsivity were −0.024, −0.014, 0.049, and 0.161, respectively, with full regression results, including coefficients, 95% confidence intervals, *p*-values, and model-fit statistics, provided in Supplementary Table S1.

To assess potential effect modification, exploratory interaction terms were introduced into the regression models. A significant interaction between BGL and sex was observed for timeliness (B = 0.014, *p* = 0.016), but not for attention (*p* = 0.226). No significant interaction between BGL and age was found (*p* = 0.126). A significant interaction between BGL and diabetes duration was observed in the conventional regression model (B = 0.024, *p* = 0.014), but this association was attenuated and did not remain statistically significant using HC3 robust standard errors. No significant interaction between BGL and HbA1c was found (*p* > 0.05).

In sex-stratified regression analyses, higher BGLs were significantly associated with higher scores in sustained attention (B = 0.014, *p* = 0.046) and timeliness (B = 0.010, *p* = 0.018) in girls. These associations remained statistically significant using HC3 robust standard errors. In boys, higher BGLs were associated with better impulsivity performance in the conventional regression model (B = 0.011, *p* = 0.049); however, this association was not statistically significant using HC3 robust standard errors (*p* = 0.062). No significant associations were observed for attention or timeliness.

## 4. Discussion

The present study examined the relationship between real-time BGL during cognitive performance in children and adolescents with T1D. The results indicate that current glycemia is associated with subtle, domain-specific variations in cognitive functioning, with effects that depend on both the analytical approach and sex.

### 4.1. Main Findings in the Context of Study Results

In unadjusted group-based analyses, children with BGLs ≥ 200 mg/dL (≥11.1 mmol/L) demonstrated nominally higher MOXO-CPT scores in sustained attention and timeliness. However, these differences did not remain statistically significant after Benjamini–Hochberg correction and were not confirmed in the adjusted regression models. No differences were observed in impulsivity or hyperactivity. Previous studies in children with T1D have generally reported poorer cognitive performance during hyperglycemia. In a field study of school-aged children with T1D, hyperglycemia was associated with longer completion times for a mental arithmetic task, while reaction time was also prolonged, although the latter finding did not reach statistical significance [19]. The discrepancy may be related to differences in study design, cognitive tasks, glucose ranges, disease duration, or other contextual factors. In addition, the present study assessed glucose at a single time point and therefore cannot determine whether the observed associations reflect glucose itself or other factors accompanying the glycemic state.

This interpretation is particularly important given that most participants in the higher-glycemia group presented with moderate rather than severe hyperglycemia. The observed pattern may nevertheless be consistent with previous reports indicating that the cognitive effects of glycemia may be domain-specific, with attentional processes and processing speed appearing particularly sensitive to acute metabolic fluctuations [1,20]. Importantly, the higher cognitive scores observed in some domains at higher BGLs should not be interpreted as evidence that hyperglycemia improves cognitive performance. Rather, these findings should be considered associations that may reflect a complex, non-linear relationship between glycemia and cognitive performance.

### 4.2. Multivariable Regression Analyses of Glycemia and Cognitive Performance

However, the unadjusted group-level differences in sustained attention and timeliness were not confirmed in the multivariable regression models after adjustment for relevant clinical and demographic covariates. The use of continuous BGL in these models provided complementary information by assessing associations across the observed glucose range, whereas the 200 mg/dL threshold was used for the prespecified group comparison. This attenuation indicates that the observed group differences were not robust to adjustment and may partly reflect differences in other characteristics between the groups. In contrast, multivariable regression analyses demonstrated that higher blood glucose levels were independently associated with higher impulsivity scores, indicating fewer impulsive responses according to the MOXO-CPT scoring system. This finding is consistent with evidence that acute metabolic states may differentially affect executive functions, including inhibitory control [21].

### 4.3. Sex as a Moderator of Glycemia–Cognition Relationship

An exploratory finding of this study was a potential sex-dependent association between glycemia and cognitive performance. A significant interaction between blood glucose levels and sex was observed for timeliness. In sex-stratified regression analyses, higher glucose levels were associated with better performance in sustained attention and timeliness in girls, and these associations remained significant using HC3 robust standard errors. In boys, the association between higher glucose levels and better impulsivity performance observed in the conventional model did not remain statistically significant using HC3 robust standard errors. These findings should therefore be considered exploratory. These exploratory findings raise the possibility that the association between glycemia and cognitive performance may differ across specific cognitive domains and between sexes.

Previous studies indicate that neurocognitive outcomes in T1D may differ between girls and boys, potentially due to differences in brain maturation, hormonal regulation, or metabolic sensitivity [6,20,22]. Pubertal hormonal changes may influence both glucose regulation and cognitive processes; however, pubertal stage and circulating sex hormone levels were not assessed in the present study. Therefore, hormonal mechanisms may represent a possible explanation for the observed sex-related differences but cannot be established from our data. Given the small size of the sex-stratified subgroups, these findings should be considered exploratory and hypothesis-generating and require confirmation in larger studies with assessment of pubertal stage and hormonal status.

### 4.4. Neurobiological Mechanisms

The observed associations may reflect several possible mechanisms, although the present cross-sectional design does not allow causal or mechanistic conclusions. Glucose is the primary energy substrate for the brain, and experimental studies suggest that acute changes in glucose availability can influence cognitive performance, although the effects may vary across cognitive domains and experimental conditions [10]. Mild hypoglycemia has been shown to reduce mental efficiency in children and adolescents with type 1 diabetes, particularly on tasks requiring attention to detail, planning and decision-making, and rapid responding [23]. In children with type 1 diabetes, naturally occurring episodes of acute hyperglycemia have been associated with reduced cognitive efficiency, particularly slower task performance, although the magnitude of these effects may vary across individuals and cognitive outcomes [19].

An alternative explanation is that BGL measured immediately before testing may partly reflect recent food intake or insulin administration, which may influence alertness and task engagement independently of glucose itself.

However, these effects appear to be domain-specific and context-dependent, which is consistent with the present findings showing selective associations across cognitive domains and potential sex-related differences.

Additionally, hyperglycemia has been associated with altered reaction time and cognitive–motor efficiency in experimental and clinical studies, although these effects may vary depending on population and context [12]. At the same time, longitudinal neuroimaging studies indicate that chronic dysglycemia is associated with structural brain changes, including alterations in white matter integrity and functional connectivity [6,9].

Together, these findings are consistent with the possibility that acute and chronic glycemic measures may relate differently to cognitive outcomes; however, the present cross-sectional data do not allow conclusions regarding the underlying mechanisms or the reversibility of these associations.

### 4.5. Acute vs. Chronic Glycemic Exposure

In the present study, HbA1c was associated with impulsivity performance but not with other cognitive domains. This suggests that chronic glycemic control may selectively influence certain aspects of executive functioning. This selective association may reflect the sensitivity of executive functions, including inhibitory control, to long-term metabolic disturbances; however, the mechanisms underlying this association cannot be determined from the present cross-sectional data. At the same time, the present findings suggest that real-time glycemia and chronic glycemic exposure may show different patterns of association with cognitive performance. This distinction highlights the dynamic nature of cognitive functioning in T1D, where acute and chronic glycemic measures may relate differently to specific aspects of cognitive performance.

An interaction between BGL and diabetes duration was observed in the conventional regression model. However, this association was attenuated and did not remain statistically significant when HC3 robust standard errors were applied. Therefore, the potential modifying effect of diabetes duration should be interpreted cautiously and considered exploratory. Although previous studies have suggested that chronic glycemic exposure and diabetes duration may be associated with differences in neurocognitive outcomes [6,24], the present data do not allow conclusions regarding adaptation, compensation, or underlying neurobiological mechanisms. Further studies with larger samples and longitudinal designs are needed to determine whether diabetes duration meaningfully modifies the relationship between real-time glycemia and cognitive performance.

In contrast, age was not associated with cognitive performance across the examined domains. This finding suggests that the observed associations were not substantially explained by age within the present cohort; however, the cross-sectional design does not allow conclusions regarding the relative contribution of developmental, disease-related, or metabolic factors.

### 4.6. Methodological Consideration

The selection of the MOXO-CPT as a measure of cognitive function represents an important methodological aspect of the present study. Continuous performance tests are widely used to assess attentional processes and executive functioning, particularly in pediatric populations, as they allow for objective and standardized evaluation of multiple cognitive domains, including attention, impulsivity, and timing [14,20,25,26,27].

In contrast to traditional neuropsychological assessments, the MOXO-CPT incorporates ecological distractors and measures response timing, providing a more comprehensive assessment of cognitive performance under conditions that better reflect real-life environments [14]. This may be particularly relevant in children with type 1 diabetes, in whom subtle and context-dependent cognitive alterations may not be captured by global cognitive measures. The use of the MOXO-CPT in the present study enabled the detection of domain-specific associations between glycemia and cognitive performance, supporting its utility as a sensitive tool for assessing cognitive functioning under varying metabolic conditions.

### 4.7. Consistency with Existing Literature

The current findings partly diverge from previous evidence on the cognitive effects of acute hyperglycemia in T1D, although the direction of these effects remains heterogeneous. Hypoglycemia has been consistently associated with cognitive impairment, whereas hyperglycemia has shown variable and context-dependent effects across different populations [12,20]. Previous studies in children with T1D have generally reported poorer cognitive performance during hyperglycemia. In contrast, in the present study, moderate hyperglycemia was associated with higher impulsivity scores in the adjusted analysis, with higher scores indicating fewer impulsive responses. However, the nominally higher sustained attention and timeliness scores observed in the higher-BGL group did not remain significant after Benjamini–Hochberg correction and were not confirmed in the adjusted regression models. These findings should therefore not be interpreted as evidence that hyperglycemia improves cognitive performance.

The direction of the present findings may be more comparable to the glucose-facilitation literature in healthy participants [10], although differences in population, metabolic status, and cognitive tasks limit direct comparison. Overall, the findings suggest that the relationship between acute glycemia and cognitive performance may be domain-specific and context-dependent.

### 4.8. Clinical Implications

These findings may have implications for the interpretation of cognitive assessments in children with T1D. Concurrent glucose levels may be worth considering when interpreting attentional assessments, particularly when testing is performed under different glycemic conditions. However, the present findings do not establish that modifying glucose levels improves cognitive performance, and they should not be interpreted as supporting higher glucose levels. Further prospective studies are needed before specific clinical or educational recommendations can be made.

### 4.9. Limitations

The present study has several limitations that should be considered when interpreting the findings. First, the cross-sectional design precludes causal inference regarding the relationship between real-time glycemia and cognitive performance. Second, blood glucose levels were assessed using a single capillary measurement immediately before testing, which does not capture glycemic variability or the direction and rate of glucose change. Therefore, the study could not determine whether cognitive performance was related to absolute glucose level, recent glucose trajectory, glycemic variability, postprandial state, or prior hypo-/hyperglycemic exposure. Third, the relatively small and unbalanced number of participants in the hyperglycemia subgroup, particularly those with markedly elevated glucose levels (>250 mg/dL), as well as in sex-stratified analyses, may have limited statistical power and generalizability in the group comparisons. The multiple comparisons across cognitive domains and subgroup analyses may have increased the risk of type I error; therefore, statistically significant findings should be interpreted cautiously and considered exploratory. Most participants in the higher glycemic group had moderately elevated glucose levels rather than severe hyperglycemia, which may limit the generalizability of the findings. Additionally, potential confounding factors such as psychosocial variables, sleep quality, or acute stress were not assessed. The study also did not include continuous glucose monitoring (CGM) data, including time in range, measures of glucose variability, and glucose trend information. These metrics could provide a more precise characterization of short-term glycemic dynamics and may influence cognitive performance independently of a single capillary glucose measurement. Finally, although the findings suggest sex-specific effects, these results should be interpreted with caution and require replication in larger, well-characterized cohorts.

### 4.10. Future Directions

Future studies should investigate the relationship between real-time glycemia and cognitive performance using continuous glucose monitoring, repeated cognitive assessments, and larger and more balanced samples. Longitudinal designs could help determine whether acute glycemic fluctuations have transient effects on specific cognitive domains and how these effects interact with diabetes duration, sex, and long-term glycemic control. Assessment of pubertal stage and hormonal status may also help clarify the sex-related differences observed in the present study.

## 5. Conclusions

In conclusion, the present study found that real-time glycemia during cognitive testing is associated with subtle, domain-specific differences in cognitive performance in children and adolescents with T1D. These associations vary across cognitive domains with exploratory analyses suggesting potential sex-related differences, highlighting the importance of considering real-time metabolic state when interpreting cognitive assessments. These findings are consistent with the possibility that cognitive performance may vary with concurrent metabolic state, although this interpretation requires confirmation in prospective studies. These findings may have clinical relevance, but their implications should be interpreted cautiously given the cross-sectional design and the relatively small hyperglycemia subgroup. The observed associations do not establish causality or indicate that higher glucose levels improve cognitive performance. This suggests that metabolic context may be worth considering when interpreting neuropsychological assessments in youth with T1D. Concurrent glucose levels may be worth considering when interpreting cognitive assessments in youth with T1D, although this should be confirmed in prospective studies before specific clinical recommendations can be made.

## Figures and Tables

**Table 1:** Clinical and demographic characteristics of patients stratified by BGL.

Variable	Group I (*n* = 93)	Group II (*n* = 24)	*p*-Value
Age [years] mean ± SD	12.9 ± 3.2	12.7 ± 3.1	0.78
Female sex *n* (%)	44 (47%)	11 (46%)	0.75
HbA1c (%) mean ± SD	7.51 ± 1.2	7.41 ± 1.1	0.74
Disease duration > 5 years *n* (%)	44 (47%)	12 (50%)	0.81
CSII treatment *n* (%)	43 (46%)	11 (46%)	0.99
BGL (mg/dL) mean ± SD	127.1 ± 33.7	236.2 ± 34.0	<0.001 *

Note: *p*, *p*-value; * *p* < 0.001; SD, standard deviation; *n*, sample size; BGL, blood glucose level; HbA1c, glycated hemoglobin; group I, BGL < 200 mg/dL (<11.1 mmo/L); group II, BGL ≥ 200 mg/dL (≥11.1 mmol/L); CSII, continuous subcutaneous insulin infusion.

**Table 2:** Comparison of MOXO-CPT parameters according to blood glucose level measured before testing.

MOXO-CPT Parameters	Group I (*n* = 93)		Group II (*n* = 24)		Statistical Values			
	Mean Value (95% CI)	SD	Mean Value (95% CI)	SD	Z	*p*	BH-Adjusted *p*	Effect Size, r (95% CI)
Sustained attention	−0.94 (−1.59 to −0.29)	3.14	0.14 (−0.51 to 0.79)	1.53	2.10	0.036	0.080	0.28 (0.10–0.44)
Timeliness	−1.11 (−1.47 to −0.75)	1.74	−0.32 (−0.32 to 0.41)	1.72	2.05	0.040	0.080	0.27 (0.09–0.43)
Hyperactivity	0.09 (−0.44 to 0.62)	2.57	0.47 (−0.39 to 1.33)	2.04	1.25	0.210	0.236	0.12 (−0.07–0.29)
Impulsivity	−1.20 (−1.87 to −0.53)	3.25	−0.31 (−1.23 to 0.61)	2.17	1.19	0.236	0.236	0.11 (−0.07–0.29)

Note: *p*, *p*-value; SD, standard deviation; Z, standardized test statistic; r, effect size; group I, BGL < 200 mg/dL (<11.1 mmol/L); group II, BGL ≥ 200 mg/dL (≥11.1 mmol/L); 95% CI, 95% confidence interval of the mean results; BH-adjusted *p*, *p*-value adjusted using the Benjamini–Hochberg procedure. Group mean values represent standardized MOXO-CPT scores (Z-scores), with higher scores indicating better performance; for impulsivity and hyperactivity, this corresponds to fewer impulsive or excessive responses.

**Table 3:** Girl-stratified analyses represent within-sex comparisons across glycemic categories.

MOXO-CPT Parameters	Girls				Statistical Values			
	Group I (*n* = 44)		Group II (*n* = 11)					
	Mean Value (95% CI)	SD	Mean Value (95% CI)	SD	Z	*p*	BH-Adjusted *p*	Effect Size, r (95% CI)
Sustained attention	−1.01 (−1.95 to −0.07)	3.09	0.82 (0.44 to 1.20)	0.56	−2.98	0.003	0.012	0.58 (0.37–0.73)
Timeliness	−1.07 (−1.61 to −0.53)	1.78	0.50 (−0.38 to 1.380)	1.31	−2.60	0.009	0.018	0.51 (0.28–0.68)
Hyperactivity	0.25 (−0.46 to 0.96)	2.32	1.01 (0.63 to 1.39)	0.56	−1.33	0.185	0.247	0.03 (−0.23–0.30)
Impulsivity	−1.06 (−2.24 to 0.12)	3.87	0.19 (−0.93 to 1.31)	1.67	−1.05	0.293	0.293	0.14 (−0.13–0.39)

Note: *p*, *p*-value; Z, standardized test statistic; SD, standard deviation; r, effect size; group I, BGL < 200 mg/dL (<11.1 mmol/L); group II, BGL ≥ 200 mg/dL (≥11.1 mmol/L); 95% CI, 95% confidence interval of the mean results; BH-adjusted *p*, *p*-value adjusted using the Benjamini–Hochberg procedure. Group mean values represent standardized MOXO-CPT scores (Z-scores), with higher scores indicating better performance.

**Table 4:** Boy-stratified analyses represent within-sex comparisons across glycemic categories.

MOXO-CPT Parameters	Boys				Statistical Values			
	Group I (*n* = 49)		Group II (*n* = 13)					
	Mean Value (95% CI)	SD	Mean Value (95% CI)	SD	Z	*p*	BH-Adjusted *p*	Effect Size, r (95% CI)
Sustained attention	−0.88 (−1.81 to 0.05)	3.23	−0.43 (−1.56 to 0.70)	1.87	−0.25	0.802	0.802	0.11 (−0.14 to 0.35)
Timeliness	−1.14 (−1.63 to −0.65)	1.72	−1.02 (−2.08 to 0.04)	1.76	−0.37	0.710	0.802	0.14 (−0.11 to 0.38)
Hyperactivity	−0.05 (−0.85 to 0.75)	2.79	0.01 (−1.62 to 1.64)	2.69	−0.42	0.672	0.802	0.01 (−0.24 to 0.26)
Impulsivity	−1.33 (−2.08 to −0.58)	2.61	−0.74 (−2.25 to 0.77)	2.50	−0.61	0.545	0.802	0.17 (−0.08 to 0.40)

Note: *p*, *p*-value; Z, standardized test statistic; SD, standard deviation; group I, BGL < 200 mg/dL (<11 mmol/L); group II, BGL ≥ 200 mg/dL (≥11.1 mmol/L); r, effect size; 95% CI, 95% confidence interval of the mean results; BH-adjusted *p*, *p*-value adjusted using the Benjamini–Hochberg procedure. Group mean values represent standardized MOXO-CPT scores (Z-scores), with higher scores indicating better performance; for impulsivity and hyperactivity, this corresponds to fewer impulsive or excessive responses.

**Table 5:** Sex differences in MOXO-CPT performance stratified by glycemic group.

	Girls		Boys		Statistical Values		
	Mean Value (95% CI)	SD	Mean Value (95% CI)	SD			
**Group I**	***n* = 44**		***n* = 49**		***p***	**BH-adjusted *p***	**Effect size, r (95% CI)**
Sustained attention	−1.01 (−1.95 to −0.07)	3.09	−0.68 (−1.52 to 0.16)	2.93	0.565	0.956	−0.06 (−0.26 to 0.15)
Timeliness	−1.07 (−1.61 to −0.53)	1.78	−1.06 (−1.55 to −0.57)	1.69	0.956	0.956	−0.01 (−0.21 to 0.20)
Hyperactivity	0.25 (−0.46 to 0.96)	2.32	−0.12 (−0.93 to 0.69)	2.83	0.769	0.956	0.03 (−0.17 to 0.23)
Impulsivity	−1.06 (−2.24 to 0.12)	3.87	−1.44 (−2.19 to −0.69)	2.62	0.178	0.712	0.14 (−0.07–0.33)
**Group II**	***n* = 11**		***n* = 13**		***p***	**BH-adjusted *p***	**Effect size, r (95% CI)**
Sustained attention	0.82 (0.44 to 1.20)	0.56	−1.10 (−3.08 to 0.88)	3.27	0.022	0.044	0.45 (0.06–0.72)
Timeliness	0.50 (−0.38 to 1.38)	1.31	−1.31 (−2.41 to −0.21)	1.82	0.008	0.032	0.52 (0.15–0.76)
Hyperactivity	1.01 (0.63 to 1.39)	0.56	0.20 (−1.33 to 1.73)	2.54	1.000	1.000	0.00 (−0.40–0.40)
Impulsivity	0.19 (−0.93 to 1.31)	1.67	−0.49 (−1.95 to 0.97)	2.41	0.378	0.504	0.18 (−0.24–0.54)

Note: *p*, *p*-value; SD, standard deviation; group I, BGL < 200 mg/dL (<11.1 mmol/L); group II, BGL ≥ 200 mg/dL (≥11.1 mmol/L); 95% CI, 95% confidence interval of the mean results; BH-adjusted *p*, *p*-value adjusted using the Benjamini–Hochberg procedure. Sex comparisons were exploratory and hypothesis-generating. For all MOXO-CPT domains, higher standardized scores indicate better performance; for impulsivity and hyperactivity, this corresponds to fewer impulsive or excessive responses.

## Data Availability

The data analyzed during this study are available upon reasonable request.

## Supplementary Materials

The following supporting information can be downloaded at: https://www.mdpi.com/article/10.3390/jcm15187080/s1, Table S1. Multivariable linear regression models for MOXO-CPT domains.
