# Supplementary material for: Association of Real-Time Glucose Levels and Cognitive Performance in Children with Type 1 Diabetes: A Cross-Sectional Study
Source: J Clin Med. 2026 Sep 12;15(18):7080. doi: 10.3390/jcm15187080 (PMC13607327; doi:10.3390/jcm15187080)
Supplement: Supplementary file 1 [file jcm-15-07080-s001.zip › jcm-4387601-supplementary.pdf]

Journal of Clinical Medicine

MDPI

Supplementary Materials

Table S1. Multivariable linear regression models for MOXO-CPT domains.

Clinical and demographic variable	MOXO-CPT domains			
	Sustained attention	Timeliness	Hyperactivity	Impulsivity
BGL	B = 0.008 (95% CI: −0.002–0.017) (p = 0.119)	B = 0.003 (95% CI: −0.003–0.009) (p = 0.322)	B = 0.006 (95% CI: −0.002–0.014) (p = 0.126)	B = 0.013 (95% CI: 0.004–0.022) (p = 0.007)
HbA1c (%)	B = 0.138 (95% CI: −0.243–0.520) (p = 0.474)	B = 0.020 (95% CI: −0.208–0.249) (p = 0.861)	B = −0.298 (95% CI: −0.609–0.012) (p = 0.060)	B = −0.653 (95% CI: −1.017–−0.289) (p < 0.001)
Age	B = 0.002 (95% CI: −0.188–0.192) (p = 0.981)	B = −0.060 (95% CI: −0.174–0.054) (p = 0.297)	B = 0.120 (95% CI: −0.034–0.275) (p = 0.126)	B = −0.039 (95% CI: −0.220–0.142) (p = 0.671)
Sex (male vs. female)	B = −0.168 (95% CI: −1.273–0.937) (p = 0.764)	B = −0.380 (95% CI: −1.042–0.281) (p = 0.257)	B = −0.294 (95% CI: −1.193–0.606) (p = 0.519)	B = −0.402 (95% CI: −1.456–0.652) (p = 0.451)
Disease duration (≤5 years vs. >5 years)	B = −0.011 (95% CI: −1.279–1.256) (p = 0.986)	B = 0.455 (95% CI: −0.304–1.214) (p = 0.238)	B = 0.838 (95% CI: −0.194–1.870) (p = 0.111)	B = 1.553 (95% CI: 0.344–2.762) (p = 0.012)
Treatment method (MDI vs. CSII)	B = 0.042 (95% CI: −1.186–1.270) (p = 0.946)	B = 0.011 (95% CI: −0.724–0.746) (p = 0.977)	B = −0.101 (95% CI: −1.101–0.899) (p = 0.843)	B = 0.094 (95% CI: −1.077–1.265) (p = 0.874)
**Model fit**	**Sustained attention**	**Timeliness**	**Hyperactivity**	**Impulsivity**
R^2	0.029	0.038	0.098	0.205
Adjusted R^2	−0.024	−0.014	0.049	0.161
F	0.551	0.731	1.997	4.722
Model p	0.768	0.626	0.072	< 0.001

Note: B, unstandardized regression coefficient; CI, confidence interval; BGL, blood glucose level; R^2, coefficient of determination; adjusted R^2, coefficient of determination adjusted for the number of predictors in the model; F, F-statistic for the overall regression model; p, p-value for the overall regression model; MDI, multiple daily injections; CSII, continuous subcutaneous insulin infusion. Diabetes duration was coded as ≤5 years = 1 and >5 years = 2. Sex and treatment method were entered as categorical predictors. Higher MOXO-CPT standardized scores indicate better performance; for impulsivity and hyperactivity, higher scores indicate fewer impulsive or excessive responses.
